# Supplementary material for: Steady-State Levels of Miro1 Linked to Phosphorylation at Serine 156 and Mitochondrial Respiration in Dopaminergic Neurons
Source: Cells. 2022 Apr 8;11(8):1269. doi: 10.3390/cells11081269 (PMC9032684; doi:10.3390/cells11081269)
Supplement: Supplementary file 1 [file cells-11-01269-s001.zip › cells-1600702_supplementary material.pdf]

cells

MDPI

Communication

Steady-State Levels of Miro1 Linked to Phosphorylation at Serine 156 and Mitochondrial Respiration in Dopaminergic Neurons

Lisa Schwarz and Julia C. Fitzgerald *

Department of Neurodegeneration, Hertie Institute for Clinical Brain Research, University of Tuebingen, 72076 Tuebingen, Germany; l.schwarz@uni-tuebingen.de

* Correspondence: julia.fitzgerald@uni-tuebingen.de

Supplementary Figure 1. Characterization of Miro1 S156A Neural Precursor cells (NPCs)

Figure S 1: (**A**) RT-qPCR showing expression of neural precursor markers *NES*, *SOX2* and *PAX6* in NPCs as fold change to iPSCs. Error bars depict SD (n=3); Wilcoxon matched pairs signed rank test with two-stage linear step-up procedure of Benjamini, Krieger and Yekutieli. (**B**) Representative immunofluorescence staining showing neural precursor marker Sox2 and Nestin in NPCs. (**C**) ECAR in hDaNs. Neurons were challenged with Oligomycin, CCCP and Rotenone with Antimycin A at the indicated times. ECAR was normalized to number of cells seeded. Error bars indicate standard deviation (n_{Diff}=3). (**D**) Quantification of intensity of Tom20, Complex IV and I Western blot bands in NPCs normalized to α-vinculin. Error bars depict standard deviation (n=4); Complex IV: Wilcoxon test matched pairs signed rank test.; Tom20 and Complex I: Paired *t* test, two-tailed. **(C)** RHOT1 knockdown in undifferentiated SH-SY5Y cells. Quantification of intensity of Miro1, Tom20, Complex IV and I Western blot bands normalized to α-vinculin. Error bars depict standard deviation (n=3); Miro1: paired *t* test, one-tailed; Tom20, Complex IV/ II/I: Paired *t* test, two-tailed. **(D)** RHOT1 knockdown in differentiated SH-SY5Y cells. Quantification of intensity of Miro1, Tom20 and Complex I Western blot bands normalized to α-vinculin. Error bars depict standard deviation (n=4); Miro1: paired *t* test, one-tailed; Tom20 and Complex I: Paired *t* test, two-tailed.

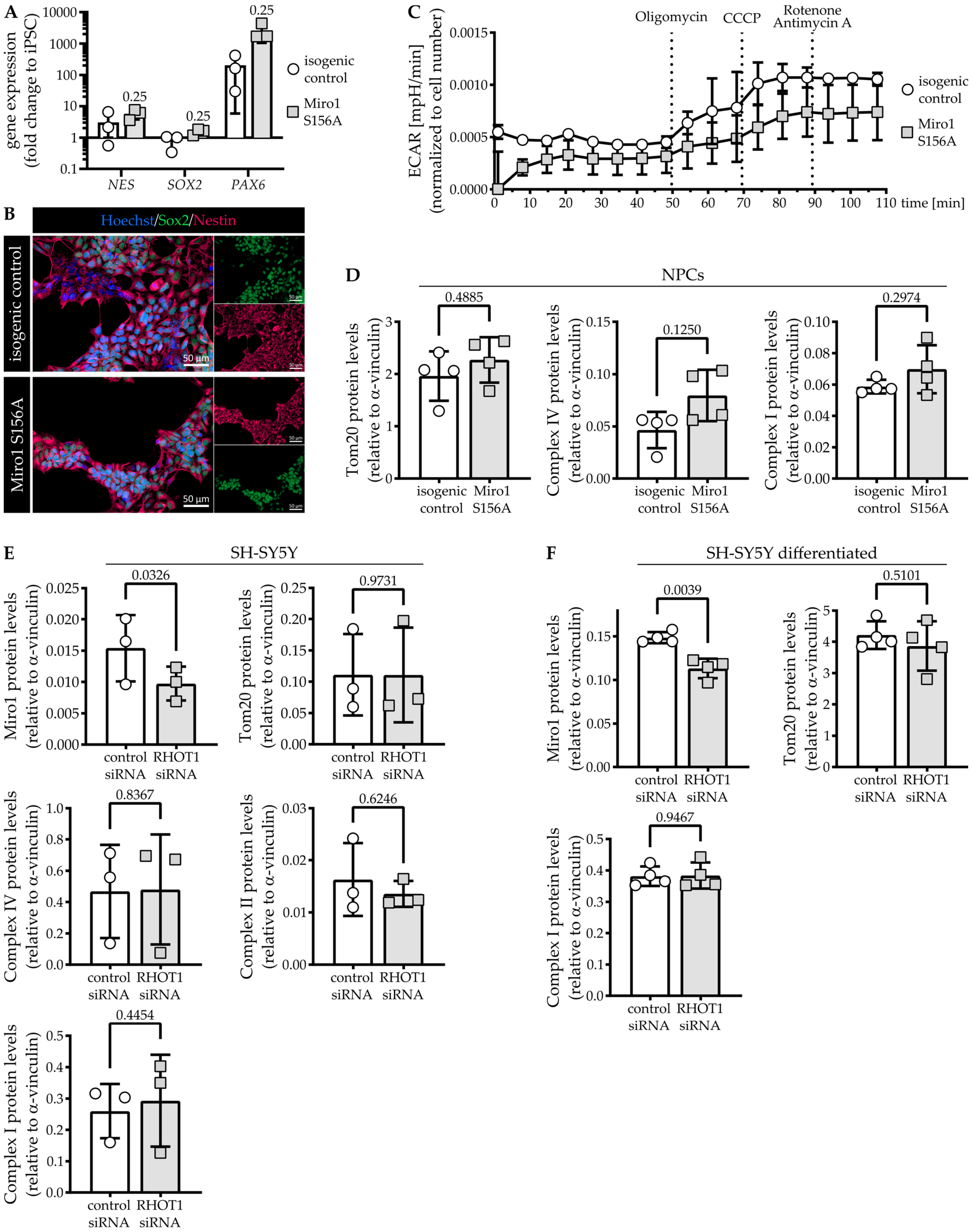
A
gene expression (fold change to iPSC)
10000
1000
100
10
1
0.1
0.25
0.25
0.25
NES
SOX2
PAX6
isogenic control
Miro1 S156A
C
ECAR [mpH/min] (normalized to cell number)
0.0015
0.0010
0.0005
0.0000
Oligomycin
CCCP
Rotenone Antimycin A
0 10 20 30 40 50 60 70 80 90 100 110 time [min]
isogenic control
Miro1 S156A
B
Hoechst/Sox2/Nestin
isogenic control
Miro1 S156A
50 µm
D
NPCs
0.4885
Tom20 protein levels (relative to α-vinculin)
0.1250
Complex IV protein levels (relative to α-vinculin)
0.2974
Complex I protein levels (relative to α-vinculin)
isogenic control
Miro1 S156A
E
SH-SY5Y
0.0326
Miro1 protein levels (relative to α-vinculin)
0.9731
Tom20 protein levels (relative to α-vinculin)
0.8367
Complex IV protein levels (relative to α-vinculin)
0.6246
Complex II protein levels (relative to α-vinculin)
0.4454
Complex I protein levels (relative to α-vinculin)
control siRNA
RHOT1 siRNA
F
SH-SY5Y differentiated
0.0039
Miro1 protein levels (relative to α-vinculin)
0.5101
Tom20 protein levels (relative to α-vinculin)
0.9467
Complex I protein levels (relative to α-vinculin)
control siRNA
RHOT1 siRNA
